# Musculoskeletal comorbidities in cardiovascular disease, diabetes and respiratory disease: the impact on activity limitations; a representative population-based study

**DOI:** 10.1186/1471-2458-11-77

**Published:** 2011-02-03

**Authors:** Morgan Slater, Anthony V Perruccio, Elizabeth M Badley

**Affiliations:** 1 Toronto Western Research Institute, University Health Network, Toronto, Canada; 2 Harvard Medical School and Brigham and Women's Hospital, Boston, USA; 3 Dalla Lana School of Public Health, University of Toronto, Toronto, Ontario, Canada

## Abstract

**Background:**

The purpose of this study was to quantify the contribution of comorbidity to activity limitations in populations with chronic cardiovascular disease, diabetes or respiratory disease (index conditions), with emphasis on musculoskeletal comorbidity (arthritis or back problems).

**Methods:**

Analysis of the 2005 Canadian Community Health Survey 3.1 (age 20+ years, n = 115,915). Prevalence ratios for activity limitations in people with the index conditions and co-occurring musculoskeletal disease, adjusted for age, gender, and socioeconomic factors, were used to estimate population associated fractions (PAF).

**Results:**

Comorbid arthritis and back problems significantly increased the risk of activity limitations across all index conditions with prevalence ratios of 1.60 and 1.46 for cardiovascular disease, 1.51 and 1.36 for diabetes, and 1.38 and 1.44 for respiratory disease for arthritis and back problems respectively. Arthritis and back problems accounted for at least 13% and 9% of activity limitations in the index populations.

**Conclusions:**

While chronic musculoskeletal conditions are not always considered priorities in chronic disease prevention, they account for a substantial proportion of activity restrictions seen in people with cardiovascular disease, diabetes and respiratory disease, with implications for prevention and control strategies.

## Background

People are living longer with chronic diseases and sedentary lifestyles and an abundance of high calorie food put people at further risk of a range of chronic conditions including heart disease, diabetes and arthritis [1,2]. The increasing burden of chronic diseases on current and future use of the health care system in industrialized countries is driving the adoption of chronic disease prevention and control strategies. A component of these strategies is helping individuals acquire the skills to manage their health and maintain an active life. A frequent focus of attention for chronic disease prevention and management programs has been on cancer, coronary heart disease, diabetes, and chronic respiratory disease with many programs focusing on a single disease[3]. However, many people have comorbidities (i.e. multiply co-occurring diseases) [4]. The occurrence of comorbidity influences decisions in many areas of health including prevention, complexity of treatment and service utilization, as well as influencing outcomes such as limitations in activity, participation restriction and mortality [5,6]. In spite of this, little attention has been paid to comorbidity and the effect that comorbid conditions have on limitations in activity and hence on one's ability to engage in self management behaviours, in particular physical activity[7,8].

Thus, the goal of this study is to quantify the contribution of comorbidity to activity limitations in people with existing chronic cardiovascular disease, diabetes or respiratory disease, with particular emphasis on musculoskeletal comorbidity, specifically arthritis and back problems, as these conditions are a frequent comorbidity [9,10] and the most frequent cause of activity limitations in the population[11,12]. Cancer was not included due to its low reported prevalence.

## Methods

Data were obtained from the 2005 Canadian Community Health Survey (CCHS, Cycle 3.1), a comprehensive cross-sectional survey of health status and determinants administered by Statistics Canada, Canada's central statistical agency. The target population included individuals 12 years of age and older living in private dwellings excluding individuals living on Indian Reserves or in certain remote areas, institutional residents and members of the Armed Forces. The survey covered approximately 98% of the Canadian population aged 12 and older.

A two-stage cluster design was employed to arrive at a representative sample of the household population. In the first stage, homogeneous strata were formed and independent samples of clusters were drawn from each stratum. In the second stage, dwelling lists were prepared for each cluster and dwellings, or households, were selected from the lists.

More specifically, each of the 10 provinces was divided into three types of regions: major urban centres, cities, and rural regions. Geographic or socio-economic strata were created within each major urban centre. Within the strata, dwellings were regrouped to create clusters.

In each stratum, six clusters were chosen by a random sampling method with a probability proportional to size, the size of which corresponded to the number of households. The other cities and rural regions of each province were stratified first on a geographical basis, then according to socio-economic characteristics. Once the new clusters were listed, the sample was obtained using a systematic sampling of dwellings. Further details of survey methodology, including strategies to ensure representativeness of the sample, have been published [13].

The questionnaire was administered using computer-assisted personal interviews (83% of the sample) and telephone interviews. With 132,221 participants, household and person response rates were high (84.9% and 92.9% respectively). For this study, analyses are focused on the adult population aged 20+ years (n = 115,915). The data used in this study were publicly available.

The presence of chronic conditions was determined using the lead-in statement: "We are interested in long-term conditions that have lasted or are expected to last six months or longer and that have been diagnosed by a health professional. Do you have...". Of the 27 specific self-reported chronic conditions that are collected by the CCHS, this study considered the following chronic conditions: cardiovascular conditions (indentified as the self-reported presence of "high blood pressure", "heart disease" or "suffer from the effects of a stroke"), "diabetes", respiratory conditions (identified as the self-reported presence of "asthma", "chronic bronchitis", "chronic obstructive pulmonary disease" or "emphysema"), "arthritis or rheumatism, excluding fibromyalgia" (referred to as arthritis), and "back problems, excluding fibromyalgia and arthritis" (referred to as back problems). All other chronic conditions were grouped together.

The health outcome of interest for this study was a global measure of activity limitation.

Prefaced with "The next few questions deal with any current limitations in your daily activities caused by a long-term health condition or problem," respondents were asked, "Do you have any difficulty...walking, climbing stairs, bending, learning or doing any similar activities?" and "Does a long-term physical condition or mental condition or health problem, reduce the amount or the kind of activity you can do...at home?...at school?...at work?...in other activities, for example, transportation or leisure?" For each of these, respondents indicated either "sometimes", "often", "never." Respondents were categorised as having an activity limitation if they responded as sometimes or often having any difficulty.

Age was grouped in 10 year intervals commencing at 20-29 to age 80+. Age over 80 years could not be further broken down as it was available in grouped format only. Race was categorised as white or visible minority. The Asian population is the largest visible minority group in Canada; only a small proportion of the population identified themselves as black or Latin American (1.7% and 0.7%, respectively). The highest level of education was dichotomized as more than high school or high school or less. Total household income was grouped as >$15,000, $15,000-$29,999, $30,000-$49,999, $50,000-%79,000, and >$80,000. Body Mass Index (BMI = weight [kg]/height [meters]^2^) was calculated using participant-reported height and weight, excluding pregnant women. BMI was considered as: underweight, (<18.5 kg/m^2^) normal weight (18.5-<24.9 kg/m^2^), and overweight/obese (≥25 kg/m^2^).

### Statistical Analyses

Data weights, provided by Statistics Canada to account for sample design, non-response and post-stratification, were used to provide estimates representative of the household population aged 20+ years. The methods employed by Statistics Canada to calculate weights have been published [13]. Variances were calculated using rescaled weights and incorporated design factors provided by Statistics Canada.

Descriptive analyses examined the distribution of demographic and socioeconomic characteristics and BMI in the overall population, and within each group reporting cardiovascular conditions, diabetes and respiratory conditions (the index conditions). Additionally, the prevalence of index and comorbid conditions, proportion with activity limitation, prevalence of comorbid conditions among the index groups, and proportion with activity limitations among pair of index and comorbid conditions were examined.

Three sets of analyses were carried out for populations defined by the presence of one of the specific chronic conditions of interest: cardiovascular conditions, diabetes or respiratory disease (index conditions). Following the methodology used in Perruccio et al,[14] adjusted prevalence ratios (PRs) for activity limitation were estimated through multivariate log-Poisson regression analyses with robust variance estimation [15,16]. The log-Poisson regression included age, gender education, income, race, BMI, and chronic conditions as indicated above. Population-associated fractions (PAF) for activity limitations were calculated for each pair of index and comorbid conditions. SAS 9.1 was used for all statistical analyses.

## Results

Table 1 contrasts the demographic and socioeconomic characteristics and BMI levels of the overall population and the groups characterized by the index conditions. The index condition groups generally were comprised of a greater proportion of older individuals; this likely partially explains some of the differences in education and income levels within these groups relative to the overall population. Nearly 51% of the overall population was overweight/obese. This is in contrast to the 74% and 67% within the diabetes and cardiovascular sub-groups, respectively.

**Table 1:** Population characteristics, overall and by index condition group.

		Overall (n = 115,915) (%)	Cardiovascular disease (n = 29,281) (%)	Diabetes (n = 8,100) (%)	Respiratory disease (n = 13,206) (%)
Age (years)					
	20-29	18.3	2.3	2.8	19.2
	30-39	18.4	4.7	4.5	15.9
	40-49	22.5	12.6	12.2	20.1
	50-59	17.8	23.2	23.5	17.0
	60-69	11.8	24.9	26.3	12.8
	70-79	7.7	21.0	21.8	10.0
	80+	3.7	11.2	8.9	5.0
Gender					
	Male	49.0	48.6	53.9	41.0
	Female	51.0	51.5	46.1	59.0
Race					
	White	83.8	87.7	85.6	88.2
	Visible minority	16.2	12.3	14.5	11.8
Education					
	High school or less	32.5	45.9	47.9	36.1
	More than high school	67.5	54.1	52.1	64.0
Income					
	<$15,000	5.8	9.2	11.1	8.9
	$15,000-$29,999	13.1	21.1	24.8	17.6
	$30,000-$49,999	20.2	23.3	23.5	19.8
	$50,000-$79,999	27.0	23.6	23.4	23.3
	>$80,000	33.9	22.8	17.2	30.4
BMI					
	Underweight	2.5	1.7	1.0	2.9
	Normal weight	46.6	31.0	25.0	42.3
	Overweight/obese	50.9	67.3	74.0	54.8

Sixty-nine percent of respondents over 20 years of age reported at least one chronic condition with 43% reporting at least one co-occurring (comorbid) condition. The prevalence of the chronic conditions and the proportion with activity limitations are presented in Table 2, along with the proportion of the population reporting pairs of index conditions with another condition and the proportion of these reporting activity limitation. Apart from the combination of cardiovascular disease with comorbid diabetes reported by 60.2%, arthritis was consistently among the most commonly reported comorbid condition, being reported by a third of people with the index conditions. The proportion of comorbid back problems was only slightly lower. Approximately half of all individuals with the index conditions reported activity limitation, the proportion being markedly higher for those with co-morbidity, particularly arthritis and back problems.

**Table 2:** Prevalence of index and comorbid conditions, proportion with activity limitation, and population-associated fractions^† ^(%) for activity limitation for index-comorbid condition pairs.

		**Chronic conditions**					
		
	**Index condition**	**CVD**	**Diabetes**	**Respiratory disease**	**Arthritis/rheumatism**	**Back problems**	**Other chronic conditions**
	
Prevalence of condition (%)		20.1	5.5	12.2	18.5	20.5	15.7
Proportion with activity limitation (%)		51.4	53.3	49.8	63.8	57.3	22.0
		Comorbid chronic conditions					
		
Proportion who have comorbid condition (%)	CVD	-	16.5	14.3	38.3	26.9	19.4
	Diabetes	60.2	-	15.3	39	25.6	8.1
	Respiratory disease	27.4	8.1	-	29.9	32.4	32.4
							
Proportion with index and comorbid condition with activity limitation (%)	CVD	-	60.7	68.9	71.3	70.4	39.3
	Diabetes	60.7	-	69.5	72.7	73.6	36.5
	Respiratory disease	68.9	69.5	-	75.3	68	30.9
							
Population Associated Fraction^† ^(%)	CVD	-	2.6	3.8	19.8	11.6	2.5
	Diabetes	13.4	-	2.9	17.8	9.4	0.8
	Respiratory disease	3.1	0	-	12.5	13.4	0

^† ^adjusted for other comorbidities, age, gender, race, education, income and BMI

Table 3 presents the association between the comorbid condition and the prevalence of activity limitations presented as prevalence ratios adjusted for age, gender, income, education, race, BMI and multimorbidity. Each column in the tables corresponds to a population of individuals with a certain index condition; the rows correspond to the factors included in the regression. Generally, the risk of activity limitations within each index condition increased with increasing age, with low income and with above- or below-average weight. Within cardiovascular disease, women (PR 0.93, 95% CI: 0.89, 0.97) and visible minorities (PR 0.85, 95% CI: 0.78, 0.93) had a lower risk of activity limitations; this association was not significant in those with diabetes or respiratory conditions. Education level was not significantly associated with activity limitations across the three index conditions. Comorbid arthritis significantly increased the risk of activity limitations across all index conditions, as did back problems, even after adjustment for other covariates.

**Table 3:** Prevalence ratios (95% CI) for activity limitation from log-Poisson regression analyses by index condition.

		Cardiovascular disease (n = 29,281)	Diabetes (n = 8,100)	Respiratory disease (n = 13,206)
Comorbid condition				
	Arthritis	**1.60 (1.53 - 1.67)**	**1.51 (1.40 - 1.62)**	**1.38 (1.30 - 1.47)**
	Back problems	**1.46 (1.41 - 1.52)**	**1.36 (1.28 - 1.45)**	**1.44 (1.36 - 1.52)**
	Cardiovascular disease	-	**1.24 (1.15 - 1.35)**	**1.09 (1.03 - 1.15)**
	Diabetes	**1.15 (1.10 - 1.20)**	-	1.00 (0.94 - 1.06)
	Respiratory disease	**1.25 (1.20 - 1.30)**	**1.17 (1.09 - 1.26)**	-
	Other chronic conditions	**1.20 (1.12 - 1.29)**	1.17 (0.97 - 1.41)	0.92 (0.83 - 1.02)
Age (years) (ref: 20-29)				
	30-39	0.79 (0.60 - 1.04)	1.79 (0.66 - 4.87)	1.11 (0.98 - 1.26)
	40-49	1.01 (0.78 - 1.29)	2.11 (0.78 - 5.68)	**1.32 (1.17 - 1.50)**
	50-59	0.97 (0.76 - 1.24)	2.04 (0.76 - 5.47)	**1.42 (1.26 - 1.60)**
	60-69	0.98 (0.77 - 1.25)	2.17 (0.81 - 5.83)	**1.47 (1.31 - 1.65)**
	70-79	1.12 (0.88 - 1.43)	2.31 (0.86 - 6.20)	**1.55 (1.37 - 1.74)**
	80+	**1.42 (1.11 - 1.81)**	**2.88 (1.07 - 7.73)**	**1.88 (1.67 - 2.12)**
Gender (ref: male)				
	Female	**0.93 (0.89 - 0.97)**	0.98 (0.92 - 1.05)	1.00 (0.95 - 1.06)
Race (ref: white)				
	Visible minority	**0.85 (0.78 - 0.93)**	0.88 (0.77 - 1.01)	0.94 (0.84 - 1.05)
Education (ref: more than high school)				
	High school or less	0.97 (0.93 - 1.01)	0.99 (0.92 - 1.05)	0.98 (0.93 - 1.04)
Income (ref: >$80,000)				
	<$15,000	**1.33 (1.23 - 1.44)**	**1.26 (1.09 - 1.46)**	**1.42 (1.29 - 1.56)**
	$15,000-$29,999	**1.23 (1.14 - 1.33)**	**1.21 (1.05 - 1.39)**	**1.30 (1.18 - 1.42)**
	$30,000-$49,999	**1.18 (1.09 - 1.27)**	1.14 (0.99 - 1.31)	**1.19 (1.08 - 1.31)**
	$50,000-$79,999	**1.12 (1.03 - 1.21)**	0.98 (0.98 - 1.14)	**1.12 (1.02 - 1.23)**
BMI (ref: normal weight)				
	Underweight	**1.23 (1.05 - 1.43)**	**1.25 (0.96 - 1.63)**	**1.27 (1.07 - 1.50)**
	Overweight/obese	**1.09 (1.04 - 1.13)**	**1.12 (1.02 - 1.22)**	**1.19 (1.12 - 1.27)**

Prevalence ratios were used to calculate population-associated fractions within each of the index condition populations for pairs of comorbid conditions (Table 2). For all three index conditions, PAFs were highest for arthritis as a comorbid condition, followed by back problems.

When arthritis and back problems were combined (results not shown), the same pattern emerged; co-existing musculoskeletal conditions increased the risk of activity limitation by at least 80%: PR 2.12, 95% CI: 2.00, 2.25 in those with cardiovascular disease; PR 1.81, 95% CI 1.66, 1.96 in those with diabetes; PR 1.86, 95% CI 1.70, 2.03 in those with respiratory disease. Co-occurring musculoskeletal disease accounted for roughly 30% of all activity limitations in people with cardiovascular disease, diabetes and respiratory disease.

## Discussion

While research has examined the impact of chronic conditions on an individual's ability to perform daily activities related to independent living,[12,17-20] this study contributes to knowledge by estimating the contribution of comorbid disease to increasing the risk of activity limitations specifically in people with existing cardiovascular disease, diabetes or respiratory disease. We found that among people with cardiovascular disease, diabetes and respiratory disease, comorbid musculoskeletal conditions (arthritis and back problems) both increased the risk and accounted for a substantial proportion of activity limitations. Comorbid cardiovascular disease also contributed a substantial proportion to activity limitations in those with diabetes.

The population associated fractions calculated in this study for populations with specific chronic diseases are generally higher that those found in an earlier study for the total population which also used activity limitations as an outcome[14]. Perruccio et al showed PAFs ranging from 3.7% to 7.5% for the contribution of diabetes and individual cardiovascular and respiratory conditions to activity limitations in the general population, adjusted for a range of comorbidities. Corresponding values for arthritis and back problems were 16.8% and 4.3% respectively. Griffith et al. [21] reported relatively large population attributable risks for functional disability (examining independently activities of daily living and instrumental activities of daily living) for pairs of chronic conditions which included arthritis. They report some of the highest values for the combination of arthritis and heart problems. Their study was limited to individuals aged 65 and over, however. These differences suggest there are differential impacts for individuals with specific medical conditions depending on the presence of particular comorbidities, and the age structure of the population under study. An important advantage of the present study was the consideration of conditions of high prevalence in the population or of considerable public interest, or both, across the full adult age range. The differential impacts we report may need to be taken into account in setting priorities for public health interventions.

Older age, lower income, and increased weight (overweight/obese) were found to be significantly associated with activity limitations in people with cardiovascular disease, diabetes and respiratory disease. This was expected as these are common risk factors for these chronic diseases[22,23] and for comorbidity[24] as well as for activity limitations and disability in general[25-27].

Arthritis, back problems, cardiovascular disease, diabetes and respiratory diseases can all interfere with physical functioning and activity levels[14,19,28]. For example, those with arthritis are not as physically active as persons without arthritis, likely partly attributable to the pain, stiffness, and joint destruction associated with the disease[29]. Studies in the US population have shown that arthritis is a barrier to physical activity such that individuals with either diabetes or heart disease who also have arthritis were more likely to be physically inactive than those with just one of these conditions[30,31]. Activity limitations can be thought of as a global measure of disability and the general definition used in the CCHS captures restrictions in numerous activities including physical function (walking, climbing stairs, etc) as well as activities at home, work, school, and leisure. This definition reflects physical performance and ability to carry out a range of activities[32] with implications for physical activity.

Our study is not without limitations as health surveys based on self-reported data have obvious disadvantages. The absence of clinician-assessed chronic conditions may lead to inaccuracies in the prevalence of chronic disease[33]. Nonetheless, such surveys are generally considered to be a reliable, economic, and practical means for measuring morbidity[33-35]. Our measure of activity limitations was a global measure and does not exclusively address physical activity as such, although this is included in the measure. As the cross-sectional nature of the data prohibits us from drawing any causal inferences, future work should build upon these findings and assess the longitudinal relationship between comorbidity and activity limitations. However, regardless of the timing of the development of the chronic conditions and outcome, the implications of musculoskeletal comorbidity on health outcomes are significant. Finally, we only considered pairs of chronic conditions and their relationship with activity limitations. Approximately one quarter of the study population reported having three or more chronic conditions and the impact of this should be investigated. Further work should also consider the impact of mental health conditions on these associations as psychological distress has been shown to add to the negative impact of chronic conditions on limitations in everyday activities[36-38].

Although numerous chronic disease prevention strategies and practice guidelines have been developed, they mainly address single chronic conditions and ignore the presence of co-existing conditions. Our findings suggest that the implementation of effective prevention, treatment and management strategies for chronic disease might be affected by musculoskeletal comorbidity. While musculoskeletal conditions are not always considered to be among the top priorities in chronic disease prevention, they are the most prevalent chronic conditions. This population-based study points to the importance of taking into account the impact of arthritis and back problems on activity limitations in persons with co-existing cardiovascular disease, diabetes and respiratory disease when designing intervention and prevention strategies.

There can be potential synergies and antagonisms in populations with comorbid conditions and potential problems in delivering intervention and prevention strategies. However, approaches which consider the presence and impact of comorbid conditions are likely of greater benefit to the population and patient than approaches which overlook them. For example, physical activity is a key prevention strategy particularly for the prevention of cardiovascular diseases and diabetes, and for health in general. When designing intervention strategies, it needs to be recognized that comorbid arthritis and back problems is not a contraindication, although modifications may need to be made [39]. Further, regular physical activity has been shown to be beneficial in managing symptoms associated with arthritis and back problems[40].

## Conclusion

The adoption of chronic disease prevention and control strategies is becoming more critical as the burden of chronic diseases in industrialized countries continues to increase. A component of these strategies encourages individuals to manage their health and maintain an active life. To date, many of these strategies have focused on single diseases. Less attention has been paid to comorbidity and the effect that comorbid conditions have on activity and consequently on one's ability to engage in self-management practices, in particular physical activity.

Among people with cardiovascular disease, diabetes and respiratory disease, comorbid musculoskeletal conditions both increased the risk and accounted for a substantial proportion of activity limitations. This suggests the implementation of effective prevention, treatment and management strategies for these chronic diseases might be affected by musculoskeletal comorbidity. While musculoskeletal conditions are not always considered to be among the top priorities in chronic disease prevention, they are the most prevalent chronic conditions. Musculoskeletal conditions and comorbidity need to be accounted for when designing intervention and prevention strategies.

## Competing interests

The authors declare that they have no competing interests.

## Authors' contributions

All authors participated in the conception and design of the study. MS acquired the data, and along with AVP performed the statistical analyses. All authors participated in the interpretation of data. MS drafted the manuscript and AVP and EMB revised it critically for important intellectual content. All authors read and approved the final manuscript.

## Pre-publication history

The pre-publication history for this paper can be accessed here:

http://www.biomedcentral.com/1471-2458/11/77/prepub
